# Operational integrity screening for telemedicine workflows: an explainable motion and audiovisual coherence framework

**DOI:** 10.3389/fbioe.2026.1828582

**Published:** 2026-06-18

**Authors:** Karol Jędrasiak, Julia Bijoch

**Affiliations:** 1 WSB University, Dabrowa Gornicza, Poland; 2 Collegium Medicum - Faculty of Medicine, WSB University, Dabrowa Gornicza, Poland

**Keywords:** audiovisual synchrony, biosecurity, digital impersonation, explainable AI, healthcare governance, motion dynamics, patient safety, telemedicine

## Abstract

Teleconsultations are exposed to digital impersonation and synthetic media attacks that can alter identity, articulation, or consent evidence, introducing emerging AI-enabled biosecurity risks to digitally mediated healthcare workflows. We evaluate an explainable integrity control based on motion dynamics and audiovisual temporal coherence, designed for conservative operation at extremely low false alarm rates with auditable evidence reporting to support governance and risk-based escalation. Our contribution is a reproducibility-oriented evaluation protocol and an evidence atlas of temporally grounded cues, together with a staged fusion that supports scalable prescreening and targeted verification under platform-style degradations characteristic of real-world dissemination chains. Building on prior biomarker-oriented analyses of physiological and structural cues for synthetic media detection, this work advances toward a calibrated, deployment-oriented integrity control architecture optimized for operational screening in telemedicine workflows. We use the DeepFake RealWorld (DFRW) dataset with 46,371 clips (229.28 h), combining 4,186 Open-Source Intelligence (OSINT) samples and 42,185 controlled, systematically degraded variants emulating recompression, resizing, filtering, and recapture. On the held-out binary-labeled test split, descriptor fusion reaches an AUC of 0.91 and achieves a true positive rate (TPR) of 18.5% (95% CI 16.2–20.8) at a false positive rate (FPR) of 0.1%, compared with 6.2% (95% CI 4.8–7.6) for an Xception baseline fine-tuned on the DFRW dataset. Microbenchmarked latencies motivate a two-stage deployment with explicit abstention and a structured integrity report aligned with healthcare governance and post-incident auditing requirements. This study evaluates telemedicine-oriented integrity controls using a benchmark dataset and does not claim demographic or clinical subgroup generalizability. Before deployment in clinical workflows, the proposed approach requires broader validation across age groups, ethnic backgrounds, speech characteristics, capture conditions, and medical conditions that may affect facial motion, articulation, or voice production. Prospective subgroup validation and governance-oriented assessment should therefore be treated as necessary directions for future work.

## Introduction

This study formalizes motion dynamics and audiovisual temporal coherence descriptors as measurable integrity controls for detecting synthetic or manipulated teleconsultation media under platform distribution and operational load. Telemedicine and teledentistry increasingly rely on clinically usable video and speech for guided self-examination, triage, and care instructions, and encounters are often treated as decision-grade evidence for documentation, prescribing, and reimbursement. We frame integrity as a testable security scenario with protected assets covering participant identity, observable symptom and examination evidence, exchanged instructions, and resulting clinical and billing records (Nieles et al., 2017). Threat actors include external fraudsters, malicious insiders, patients, third parties, and opportunistic attackers leveraging publicly available face or voice material; feasible vectors include face reenactment, face swap, full-frame generative synthesis, voice cloning, lip synchronization manipulation, selective editing, and replay of previously recorded authentic segments. Attacks can induce clinically unsafe decisions or unauthorized administrative actions such as prescriptions, referrals, certificates, or reimbursements, with harms including inappropriate reassurance, delayed escalation, inappropriate medication or follow-up, corrupted documentation, fraud loss, incident response workload, and erosion of trust. We define attacker success as the completion of a high-impact workflow step using synthetic or materially manipulated audiovisual content and defender success as detection or reliable suspicion at a pre-specified low false alarm level that triggers escalation before action finalization, supported by an auditable evidence rationale. The operational goal is early risk identification for additional verification steps, not universal binary authentication. Table 1 maps minimum risk scenarios and controls to teleconsultation workflow steps, linking process points to measurable signals and procedural controls to support a verifiable deployment narrative.

**TABLE 1:** Teleconsultation threat model mapping of risks to workflow steps and candidate controls.

Risk scenario	Workflow step	Adversary and vector	Candidate controls and success criterion
Patient impersonation	Identity, consent, prescribing	External fraudster or third party; face swap, reenactment, voice cloning, replay	Step-up identity checks, 2FA, challenge response, liveness; cues E_Δ, motion boundary histograms (MBH), LogSumExp (LSE) D, Δt_AV, J; success: flag before prescribing with low false alarm burden
Clinician impersonation	Instructions, consent capture, care plan	External attacker or insider; voice cloning, synthetic video, account hijack	Secure session plus provenance, identity verification, a second-channel for high risk; cues LSE D, Δt_AV; success: verify before patient action or consent recording
Visual symptom manipulation	Visual examination, triage	Patient or third party; local edits, inpainting, reenactment	E_warp and flow consistency, MBH, controlled capture prompts; success: inconsistencies trigger recapture or escalation
Instruction manipulation	Treatment plan, medication instructions	External attacker or insider; edited audio, synthetic speech, timing manipulation	Provenance or signing, second-channel confirmation; cues Δt_AV, r_F0M, LSE D; success: block or confirm before action
Billing abuse	Billing, payer documentation	Fraudster or collusion; impersonation, falsified segments	Audit trail, identity binding, metadata cross checks, plus motion and synchrony cues; success: reduce fraud with low false-positive burden

Teleconsultation media exhibit strong distribution shifts due to recompression, bitrate adaptation, resolution changes, augmented reality filtering, and recapture, which can suppress frame-level artifacts and degrade detectors outside their training distribution. Risk reduction guidance, therefore, stresses the evaluation of the target content distribution and realistic dataset design. We adopt the working hypothesis that motion dynamics and audiovisual temporal coherence are more stable and explainable under platform conditions than purely spatial cues because generation pipelines can optimize frame-level realism while failing to guarantee physically plausible temporal evolution, yielding abnormal micro-rhythm, nonphysical smoothness or jerkiness, inconsistent warping residuals in nonrigid facial regions, or phoneme viseme desynchronization. These signals can be localized and quantified as kinematic or audio-to-mouth timing deviations to support defensible escalation decisions in clinical governance and security operations. To enable systematic evaluation in the contemporary diffusion era and amid repeated platform transformations, we develop the DeepFake RealWorld dataset (DFRW), combining OSINT samples with generated and systematically degraded variants that emulate distribution chains. The DFRW dataset contains 46,371 clips (229.28 h), including 4,186 OSINT samples and 42,185 generated or transformed variants spanning reenactment, face swap, and diffusion-based video generation. The main contributions are an empirically validated atlas of motion and temporal descriptors selected under operational thresholds (p_df ≥ 30%, p_real ≤ 20%, and either Δp ≥ 0.15 or precision–recall (PR) ≥ 1.5; the p_df ≥ 30% threshold applies to motion and flow descriptors at the p_real ≤ 20% operating point, whereas audiovisual descriptors at a fixed extremely low FPR are admitted via high PR and low-FPR sensitivity), with stability defined as a relative drop ≤ 0.15, that is, ≤ 15%, under explicit degradation chains to reflect low false alarm escalation; a composite demonstrating complementarity of motion and audiovisual synchrony for scalable prescreening and targeted verification; and an evidence reporting path aligned with calibrated decision support and auditable deployment.

### Related work

Telemedicine and teledentistry are now routine channels for access, triage, follow-up, and patient education. Telehealth guidance for oral health emphasizes reliable audio and video, patient preparation, and workflows that enable clinically adequate remote assessment of the intraoral and facial area (Telehealth.HHS.gov, 2024). Dental policy statements position teledentistry as an adjunct to in-person care and distinguish synchronous encounters from asynchronous store-and-forward modalities that require explicit documentation of what was captured, how it was transmitted, and how it is retained (American Dental Association, 2023; American Academy of Pediatric Dentistry, 2025). Empirical evidence indicates that telemedical diagnostic concordance and clinician confidence depend on modality completeness and signal quality, motivating signal quality auditing alongside evidence integrity (Demaerschalk et al., 2022; Krupinski et al., 1999; Moreira, 2025).

Healthcare-focused discussions of deepfakes highlight low-cost impersonation and fabricated authority cues, but the teleconsultation problem is more direct: manipulation of identity or encounter content can alter clinical decisions, documentation integrity, and downstream safety (Jędrasiak and Bijoch, 2026). Many media forensics detectors rely on photometric or frame-level cues and often degrade under the distribution shifts caused by generator changes, recompression, recapture, resolution reduction, and platform transcoding (Li et al., 2018). Operational settings further require very low false alarm rates, robustness to unknown quality, and explainable evidence suitable for auditing because false alarms impose workflow cost while false negatives may enable harmful impersonation or content manipulation.

Temporal modeling and multimodal analysis have therefore become central for improving robustness under platform processing. Motion dynamics, flow regularities, and audiovisual timing relations can remain informative when fine-grained spatial artifacts are attenuated, and they support evidence narratives based on measurable deviations in kinematic coherence or audio-to-mouth timing rather than opaque scores. Benchmarking remains the main bottleneck for such claims (Lu and Ebrahimi, 2022; Lu and Ebrahimi, 2024) (Lu and Ebrahimi, 2022; Lu and Ebrahimi, 2024). DFDC (Dolhansky et al., 2020) increased diversity and scale but reflects earlier generator regimes and was not designed for systematic evaluation of audiovisual synchrony or long-horizon temporal cues. FaceForensics++ (Rössler et al., 2019) enables controlled ablations but offers limited representativeness of repeated platform transcodes, recaptures, and broader motion regimes. Celeb DF v2 (Li et al., 2020) improves photorealism and highlights generalization gaps but still only partially supports systematic assessment of temporal and timing metrics. DeeperForensics 1.0 (Jiang et al., 2020) moves toward distribution realism via degradations and controlled compression but remains anchored in earlier generator families and does not fully reflect modern diffusion-based and hybrid systems. These limitations align with the broader risk management position in NIST guidance (NIST, 2024) on synthetic content, which emphasizes evaluation under realistic conditions and the centrality of distribution-relevant testing when building detection and transparency mechanisms. This motivates the DFRW dataset and the associated protocol, which emulate platform distribution chains to support descriptor-level assessment under explicit operational constraints.

## Materials and methods

### Dataset and protocol

The DeepFake RealWorld dataset (DFRW) targets distribution-realistic evaluation of motion dynamics and audiovisual temporal coherence under platform processing, with explicit support for conservative operation at low false alarm rates. The DFRW dataset contains 46,371 video clips (229.28 h), including 4,186 OSINT clips (9%) and 42,185 controlled, generated, or systematically transformed clips (91%). Manipulation coverage includes contemporary families such as diffusion-based synthesis and hybrid pipelines, while dissemination is emulated through logged degradations and recaptures.

Annotations decouple the semantic label y from label confidence c. Labels follow y∈{Real, Manipulated}. Real indicates no supported material manipulation under the verification protocol. Manipulated covers generation or edits that alter identity, articulation, or visual evidence, including face swap, reenactment, audio-driven lip synchronization, full-frame diffusion synthesis, and complex edits. Confidence follows c∈{High, Medium, Low}. High applies to deterministically known controlled logs or OSINT corroborated by multiple independent sources, plus consistency checks. Medium applies to partially corroborated OSINT. Low applies to weak or conflicting OSINT evidence. Clips with c = Low are excluded from supervised training and headline operating point reporting but are retained for stress testing and qualitative failure analysis. OSINT curation follows harm minimization, excludes privacy-violating content for nonpublic individuals and explicit sexual content, and minimizes the storage of identifiers. Near duplicates are detected before splitting using perceptual hashing with a Hamming distance ≤10. On the pre-removal pool, this corresponds to a 1.3% duplicate rate, and all detected duplicate groups are removed prior to splitting, yielding a retained set with a near-duplicate rate of approximately 0 under the same criterion.

The controlled subset provides fully logged generation and transformation parameters, enabling high-confidence labels by construction. Across the full DFRW corpus (N = 46,371), manipulation families include 35% face swap (16,230), 28% reenactment and expression manipulation (12,984), 25% full-frame diffusion synthesis (11,593), and 12% complex scene manipulations (5,564). Within the controlled subset (N = 42,185), per-family counts and shares are reported in the immutable release manifest and sum to 42,185. Diffusion logs record sampling steps and guidance scale (median steps 30, median guidance 4.5). Reenactment is annotated as audio-driven (57%) or video-driven (43%). Authentic Real material for false alarm evaluation comes from OSINT verified as authentic and from controlled authentic sources used as transformation inputs when licensing and privacy allow. In the binary-labeled portion of the DFRW dataset, Real totals 20,000 clips, including 1,800 verified OSINT clips and 18,200 controlled authentic source clips, where the 18,200 clips are included in the controlled subset total of N = 42,185. To align distributions, matched degradation chains are applied to the Real and Manipulated material. When the raw, authentic sources cannot be redistributed, the release provides derived descriptors and cryptographic hashes.

Platform distribution is emulated via auditable degradation chain records per clip, including repeated AVC H.264 and HEVC H.265 reencoding (CRF 18–32), resolution downscaling, aspect ratio changes, mobile style filtering, augmented reality overlays, watermarks, and recapture. The mean number of reencodes is 2.1, with 36% one-pass, 31% two-pass, 23% three-pass, and the remaining 10% four- (or more) pass encodings. Mobile and augmented reality filters occur in 28% of clips and watermarks in 19%. Degradation strata are 15% high quality (6,956), 60% medium degradation (27,823), and 25% high degradation (11,592). Codecs are 71% AVC H.264, 19% HEVC H.265, 7% VP9, and 3% AV1. The median bitrate is 1.6 Mb/s (IQR 0.9 Mb/s to 2.8 Mb/s). Resolutions are 14% 480p or lower, 45% 720p, 33% 1080p, and 8% 1440p or higher. Frame rate distribution includes 62% 30fps, 21% 24fps, 7% 60fps, 7% 25fps, and 3% other. Quality strata are validated with NIQE medians 2.6, 3.2, 4.1 and BRISQUE 24, 31, 42 for high quality, medium degradation, and high degradation, respectively (Mittal et al., 2012a; Mittal et al., 2012b). CRF correlates with perceptual metrics (ρ = 0.82 for CRF *versus* LPIPS, ρ = −0.79 for CRF *versus* VMAF, p < 0.001), where LPIPS follows Zhang et al. (2018), and VMAF follows Bampis et al. (2018). For the subset with reference sources, the PSNR is 33.8 dB, SSIM 0.926, and LPIPS 0.193 (Wang et al., 2004). Clip duration is 17.8 s ± 12.3 s (95% CI (17.69 s, 17.91 s)), median 12.4 s (IQR 7.1 s–22.9 s), with 22% <5 s (10,202), 41% 5 s–15 s (19,012), 27% 15 s–60 s (12,520), and 10% >60 s (4,637). Audio is present in 77% of clips, with 58% 44.1 kHz and 40% 48 kHz sampling rates, and 63% mono versus 37% stereo. Each instance stores SHA256 hashes, technical metadata, generation or transformation logs, degradation chain records, and annotation versioning.

Splits are 70% training, 15% validation, and 15% test, stratified by manipulation family, degradation level, clip length, and frame rate, with enforced identity separation. OpenFace embedding clustering (Amos et al., 2016) indicates 8,740 ± 210 unique identities on the subset of clips for which a valid face embedding is available. On this embedding-available subset, the mean cluster size is 3.2, and the median is 2. Therefore, these cluster statistics are not expected to sum to the full corpus size of 46,371 clips. A leakage test based on cosine distance < 0.3 reports 0% cases. Descriptor selection follows conservative constraints using p_df ≥ 30%, p_real≤20%, and either Δp ≥ 0.15 or PR ≥ 1.5, with stability defined as a relative drop ≤ 0.15, that is ≤ 15%, under specified degradation chains. The p_df ≥ 30% threshold applies to motion and flow descriptors at the p_real ≤ 20% operating point; audiovisual descriptors evaluated at a fixed extremely low FPR (e.g., Δt_AV at p_real = 5.1%) are admitted via high PR and low-FPR sensitivity rather than the p_df ≥ 30% rule. See the Table 4 note (r_F0M / Δt_AV) for the full explanation. The present evaluation should not be interpreted as demographic or clinical subgroup validation. The DFRW dataset was designed primarily as a distribution-realistic benchmark for synthetic media integrity screening, rather than as a clinically enrolled telemedicine cohort. Consequently, demographic and medical status variables are not available with sufficient completeness, consistency, and legal certainty to support definitive subgroup-level claims. This limitation is important because detector performance may plausibly vary according to age, ethnic background, skin tone, facial morphology, speech characteristics, language, recording device, lighting conditions, and medical conditions affecting facial motion, articulation, voice production, or head movement. Relevant examples include facial palsy, neuromuscular disorders, dysarthria, tremors, dental or craniofacial abnormalities, respiratory impairment, and post-surgical facial changes, all of which may influence the motion and audiovisual synchrony cues used by the proposed system. For this reason, demographic and clinical subgroup generalization is treated as an explicit limitation of the present study, rather than as an assumed property of the model.

Availability is versioned via an immutable manifest per release containing clip_id, provenance type, modality flags, license restriction flags, degradation chain identifiers, and SHA256 hashes. Reproducibility is therefore qualified rather than claimed as unrestricted end-to-end replication. For non-redistributable OSINT, reproducibility is provided at the descriptor and evaluation level via manifests and hashes, exported derived descriptors and intermediate features with extraction code versioning, deterministic degradation scripts for controlled sources, identity cluster IDs for splits, and an evaluation package reproducing metric computation, threshold selection, and reporting. Exact end-to-end replication on identical raw OSINT media is not claimed when the original assets cannot be redistributed because of third-party terms, privacy constraints, platform restrictions, or ethical data minimization requirements. The intended reproducibility target is thus protocol-level and metric-level verification, not unrestricted reconstruction of every raw media input. Flow of clips and evaluation subsets follow y∈{Real, Manipulated} with confidence c∈{High, Medium, Low}. Supervised training and headline operating point reporting use only y∈{Real, Manipulated}. Audiovisual cues are reported only when audio is present, and speech is usable according to the preprocessing criteria defined in the Materials and Methods section using U_speech, yielding 5,356 test clips (2,310 real and 3,046 manipulated), which is 77% of the binary-labeled test split.

We define reproducible motion and audiovisual temporal coherence descriptors motivated by the hypothesis that generators optimize frame-level realism without enforcing physically plausible temporal evolution, yielding anomalous micro-rhythm, unstable or over-smoothed motion fields, and audio-to-articulation mismatches. Clips are decoded with ffmpeg (FFmpeg Developers, 2024) into RGB frames and PCM audio. Scores are normalized to per second by FPS. FPS>60 is temporally subsampled to 60fps; FPS < 20 flags second-derivative-based descriptors as low reliability. Face processing uses frames resized so the shorter side is 720px. Optical flow is computed on BT.601 luminance Y (ITU-R, 2011). Faces are detected per frame with RetinaFace ResNet50 (Deng et al., 2020) using a score ≥0.80 and NMS IoU 0.40. The largest face is selected, and multi-face clips with near equal areas (within 15%) are excluded from ROI reporting unless explicitly analyzed. Tracking uses IoU association with a maximum center displacement of 0.25 box diagonal per frame; >10 consecutive failures flag unstable tracking. Landmarks use MediaPipe Face Mesh (468) (Lugaresi et al., 2019) with tracking confidence ≥0.70, Savitzky–Golay smoothing (11 frames, order 2) (Savitzky and Golay, 1964) after MAD outlier removal (3.5), and linear interpolation for gaps ≤5 frames. Larger gaps trigger abstention for mouth or eye geometry-dependent descriptors. Semantic masks use BiSeNet face parsing (Yu et al., 2018) on a 512 × 512 aligned crop (CelebAMask HQ), threshold 0.50 plus morphological closing (5px), defining lips, eyes, jawline, mid-face, and background ROIs. ROI stabilization uses the alignment transform to reduce drift. Clips are excluded from descriptor reporting when face width <120px, Laplacian variance <80, occlusion exceeds 30% relative to the temporal median, or >20% frames miss detection or landmarks. AR overlays covering >25% of the lips ROI are flagged and analyzed separately. All exclusions are logged, and per descriptor retained counts are reported.

We compute flow in classical regimes that tolerate severe compression and in a deep regime for a higher fidelity reference. Farnebäck dense flow uses OpenCV defaults with pyr_scale 0.50, levels 5, winsize 21, iterations 5, poly_n 7, poly_sigma 1.50, and Gaussian pre-smoothing σ = 1.00. Lucas–Kanade tracks sparse points with winSize 21 × 21, maxLevel 3, termination 30 iterations or ε = 0.01, discarding points with forward-backward error >1.5px. Recurrent all-pairs face transforms (RAFTs) run on aligned face crops (Teed and Deng, 2020) (20 update iterations) and are computed only when face width ≥160px and bitrate >0.5 Mb/s; otherwise, RAFT descriptors are marked low reliability.

Descriptors are computed per frame or frame pair and aggregated over 2.0 s windows with 1.0 s stride using robust statistics. The clip score is the maximum window 90th percentile to capture localized manipulations. We report interframe difference energy E_Δ as mean squared Laplacian filtered luminance differences within ROIs (lips emphasized); motion boundary histograms (MBH) from Sobel gradients of flow with eight bins over spatiotemporal cuboids, scored by χ^2^ distance to an authentic validation reference per degradation stratum; forward-backward flow consistency error E_fb within face ROI; cross-regime end-point error (EPE) between RAFT and Farnebäck when RAFT reliability holds; jerkiness J as variance of the second temporal derivative of median flow magnitude normalized by its median; warping residual energy E_warp as the mean squared deviation between the observed flow and the rigid motion predicted from the perspective-n-point (PnP) head pose using landmarks, emphasized near the jawline and hairline-adjacent regions; background-to-face motion ratio R_bg_face as deviation from the authentic validation median ratio per degradation stratum; and optional bitstream motion cues when codec motion vectors are available.

Audio is resampled to 16 kHz mono and normalized to −23LUFS following EBU R128 and Tech 3341 (EBU, 2023a; EBU, 2023b). WebRTC VAD (WebRTC Project Authors, 2015) detects speech and defines U_speech as speech fraction times an SNR proxy; U_speech<0.10 yields speech-absent, disabling audiovisual metrics. Because U_speech can be reduced intentionally by speech suppression or music masking, modality absence is logged and can be treated as a risk context for escalation or guided recapture because audiovisual fusion provides higher operational PR and Δp than motion-only cues at matched low false positive rates (FPRs). Mouth opening is computed from inner lip landmarks and normalized by inter-ocular distance, with 0.20 s median filtering. Lip sync metrics LSE C and LSE D follow SyncNet style embeddings as in Wav2Lip (Prajwal et al., 2020) using 96 × 96 mouth crops and matched 0.20 s audio segments. Scores are computed only on speech segments and are null for speech-absent clips. An audiovisual delay estimate Δt_AV is obtained by maximizing cross-correlation between the mouth-opening derivative and audio energy envelope over lags −0.50 s to +0.50 s in 0.01 s steps, set to null for speech-absent or music-dominant audio. Pitch mouth coupling uses YIN F0 estimation (de Cheveigné and Kawahara, 2002) on voiced frames, correlating log F0 with mouth motion derivative per window. The reported score is r_F0M = 1−median_w|ρ_w|, with null assignment when speech is insufficient, or F0 tracking is unreliable. A phoneme viseme alignment error e_PV is computed only when language identification and forced alignment are available; otherwise, it is omitted, making language dependence explicit.

For each descriptor d, we compute a clip score s_d with unified polarity, so “higher” indicates stronger manipulation evidence. At threshold τ, p_df(d,τ) is the exceedance rate on Manipulated clips and p_real(d,τ) is the exceedance rate on Real clips, computed on the descriptor eligible subset after modality availability, speech usability, and ROI quality filters. These correspond to the conditional true positive rate (TPR) and the conditional FPR given eligibility E_d, with denominators reported as Denom A or Denom B in Tables 2–5. Δp(d,τ) = p_df−p_real and PR(d,τ) = p_df/max(p_real, 1/N_real), where 1/N_real is a finite sample floor that prevents undefined or spuriously infinite ratios when the observed exceedance count on Real clips is zero at a given τ. Operationally, PR summarizes lift at the operating point as the expected number of manipulated exceedances per one authentic exceedance under equal class priors, and it can be mapped to workload using Esc_per_1000 under deployment base rates. Thresholds are parameterized by target α∈{0.01, …, 0.30}, using τ_{d,α} as the (1−α) quantile of s_d on Real validation so p_real≈α, then selecting α_d* maximizing Δp for α ≤ 0.30 and reporting τ_{d,α_d*} on test. Atlas inclusion requires p_df ≥ 30%, p_real≤20%, and either Δp≥0.15 or PR ≥ 1.5 at the operating point, plus stability defined as a relative performance drop of at most 0.15, that is, at most 15%, under defined degradation chains. The p_df ≥ 30% threshold applies to motion and flow descriptors at the p_real ≤ 20% operating point; audiovisual descriptors evaluated at a fixed extremely low FPR (e.g., Δt_AV at p_real = 5.1%) are admitted via high PR and low-FPR sensitivity rather than the p_df ≥ 30% rule. See the Table 4 note (r_F0M / Δt_AV) for the full explanation. We evaluate spatial, temporal, and audiovisual baselines on identical DFRW train, validation, and held-out test splits with matched strata. For Xception, we report a model trained on FaceForensics++ and evaluated zero-shot on the DFRW dataset and a tuned variant initialized from the FaceForensics++ checkpoint and fine-tuned on the DFRW training split using binary labels, with early stopping on the DFRW validation split. For the tuned baseline, operating thresholds are selected on the same validation split to match the target low false positive rates used for headline reporting in Table 5. Frames are sampled at 1fps using 299 × 299 face crops, and clip-level scores are computed as the mean of frame log odds. We further include an I3D style temporal model initialized from Kinetics 400 and adapted on FaceForensics++ (Carreira and Zisserman, 2017) and a SyncNet-based discriminator obtained by logistic calibration of LSE C and LSE D on validation. Baselines are compared using AUC and operational metrics at matched extremely low false alarm levels, with the atlas framed as conservative, auditable escalation support with explicit abstention rather than universal authentication.

**TABLE 2:** Flow of clips and evaluation subsets in the DFRW dataset, including modality-dependent filters.

Subset or stage	Real	DF	Total	Share	Notes
Full DFRW corpus (binary labels)	20,000	26,371	46,371	100.0%	Includes low confidence OSINT (c = Low); excluded from supervised training and headline metrics
OSINT collected sources	1,800	2,386	4,186	9.0%	Provenance uncertainty: raw redistribution may be restricted
Controlled generation and logged transformations	18,200	23,985	42,185	91.0%	Deterministic label from logs includes systematic degradations
Binary-labeled test split (headline reporting)	3,000	3,956	6,956	15.0% of the full corpus	Evaluation denominators in tables and figures
Speech-usable test subset (audiovisual cues)	2,310	3,046	5,356	77.0% of the test	Audio present and U_speech≥0.10; used for LSE and Δt_AV
Descriptor-specific ROI and modality filters	Varies	Varies	Varies	Varies	Counts vary by descriptor; retained counts are in Tables 3, 4

**TABLE 3:** Unconstrained regime.

Desc	Fam	α*/τ	FPR/TPR	Δp	PR	Nset
E_Δ	Mot	0.28/0.42	28.1%/54.2%	0.26	1.93	A
MBH	Flow	0.30/1.85	30.0%/54.9%	0.25	1.83	A
E_warp	Flow	0.30/0.18	29.8%/53.6%	0.24	1.80	A
J	Mot	0.25/0.09	25.4%/40.1%	0.15	1.58	A
LSE D	AV	0.20/7.10	20.0%/44.0%	0.24	2.20	B
Δt_AV	AV	0.05/0.12	5.1%/21.4%	0.16	4.20	B
r_F0M	AV	0.22/0.65	22.0%/39.6%	0.18	1.80	B
Xception	Base S	0.15/0.55	15.2%/68.4%	0.53	4.50	A
I3D	Base T	0.20/0.48	20.5%/51.2%	0.31	2.50	A
SyncNet	Base AV	0.20/0.50	19.8%/41.5%	0.22	2.10	B

Operating points and test split subset counts. p_real = FPR, and p_df = TPR. α* is selected on validation, and τ is the corresponding validation quantile threshold. Counts are stratified by HQ, MD, and HD. Denom denotes the denominator set used for the reported counts, where A is the full binary-labeled test split, and B is the speech-usable subset with audio present and U_speech ≥0.10. A: Real N = 3,000 (HQ 450, MD 1800, HD 750), DF N = 3,956 (HQ 593, MD 2374, HD 989). B: Real N = 2,310 (HQ 346, MD 1386, HD 578), DF N = 3,046 (HQ 457, MD 1828, HD 761).

**TABLE 4:** Constrained operating point regime with p_real ≤20% enforced on validation; atlas inclusion additionally requires p_df≥30% and either Δp≥0.15 or PR≥1.5: operating points and test split subset counts.

Descriptor	Family	α*/τ (val)	p_real/p_df (test)	Δp	PR	Denom
E_Δ	Motion	0.20/0.48	19.8%/41.6%	0.22	2.10	A
MBH	Flow	0.20/2.10	20.1%/40.8%	0.21	2.03	A
E_warp	Flow	0.20/0.22	19.5%/38.0%	0.19	1.95	A
J	Motion	0.20/0.12	19.9%/32.8%	0.13	1.65	A
LSE D	Audiovisual	0.20/7.10	20.0%/44.0%	0.24	2.20	B
Δt_AV	Audiovisual	0.05/0.12	5.1%/21.4%	0.16	4.20	B
r_F0M	Audiovisual	0.15/0.72	15.0%/27.0%	0.12	1.80	B
Xception	Baseline spatial	0.10/0.65	10.2%/58.1%	0.48	5.70	A
I3D	Baseline temporal	0.15/0.55	15.1%/42.3%	0.27	2.80	A
SyncNet	Baseline AV	0.15/0.60	14.8%/31.1%	0.16	2.10	B

p_real = FPR, and p_df = TPR. τ is chosen on validation, and all metrics are recomputed on the held-out test split. For each descriptor, α∗_d is selected on validation to maximize Δp within the constrained range (p_real ≤ 20%); the chosen operating point may therefore lie below 20% when a lower FPR yields the best separation (e.g., Δt_AV at 5.1%). Counts are stratified by HQ, MD, and HD. A: Real N = 3,000 (HQ 450, MD 1800, HD 750), DF N = 3,956 (HQ 593, MD 2374, HD 989). B: Real N = 2,310 (HQ 346, MD 1386, HD 578), DF N = 3,046 (HQ 457, MD 1828, HD 761). Because τ is fixed on validation, p_real on the held-out test split can slightly exceed 20% due to sampling variability (e.g., MBH at 20.1%). This does not violate the validation constraint. Denom A refers to the full binary-labeled test split, and Denom B refers to the speech-usable subset with audio present and U_speech ≥0.10.

**TABLE 5:** Standard curve metrics and fixed-FPR operating points on the DFRW test split (95% confidence intervals).

Method	AUC (95% CI)	pAUC FPR≤1% (95% CI)	pAUC FPR≤0.1% (95% CI)	TPR at FPR = 1% (95% CI)	TPR at FPR = 0.1% (95% CI)	Test subset
E_Δ	0.78 (0.76–0.80)	0.09 (0.08–0.10)	0.005 (0.004–0.006)	12.4% (11.0–13.8)	3.2% (2.1–4.3)	Binary-labeled clips
MBH	0.76 (0.74–0.78)	0.08 (0.07–0.09)	0.004 (0.003–0.005)	10.8% (9.5–12.1)	2.8% (1.9–3.7)	Binary-labeled clips
E_warp	0.75 (0.73–0.77)	0.08 (0.07–0.09)	0.004 (0.003–0.005)	11.2% (9.8–12.6)	2.5% (1.5–3.5)	Binary-labeled clips
J	0.68 (0.66–0.70)	0.04 (0.03–0.05)	0.001 (0.000–0.002)	5.5% (4.2–6.8)	0.9% (0.4–1.4)	Binary-labeled clips
LSE D	0.81 (0.79–0.83)	0.11 (0.10–0.12)	0.006 (0.005–0.007)	14.5% (13.0–16.0)	4.1% (3.0–5.2)	Speech-usable subset
Δt_AV	0.79 (0.77–0.81)	0.13 (0.11–0.15)	0.009 (0.007–0.011)	18.2% (16.5–19.9)	7.5% (6.1–8.9)	Speech-usable subset
Xception tuned	0.88 (0.86–0.90)	0.18 (0.16–0.20)	0.008 (0.006–0.010)	22.5% (20.8–24.2)	6.2% (4.8–7.6)	Binary-labeled clips
r_F0M	0.72 (0.70–0.74)	0.05 (0.04–0.06)	0.002 (0.001–0.003)	6.8% (5.5–8.1)	1.5% (0.8–2.2)	Speech-usable subset
Xception FF++	0.65 (0.63–0.67)	0.02 (0.01–0.03)	0.000 (0.000–0.001)	3.1% (2.2–4.0)	0.3% (0.0–0.6)	Binary-labeled clips
I3D off the shelf	0.74 (0.72–0.76)	0.06 (0.05–0.07)	0.002 (0.001–0.003)	8.2% (7.0–9.4)	1.8% (1.0–2.6)	Binary-labeled clips
SyncNet	0.77 (0.75–0.79)	0.07 (0.06–0.08)	0.003 (0.002–0.004)	9.5% (8.2–10.8)	2.1% (1.2–3.0)	Audio-present subset
Fusion S_pre + S_ver	0.91 (0.89–0.93)	0.35 (0.32–0.38)	0.025 (0.021–0.029)	42.0% (39.5–44.5)	18.5% (16.2–20.8)	Binary-labeled clips

Confidence intervals are computed by nonparametric bootstrap with stratification as described in the Results section. pAUC is computed on the restricted FPR interval. For consistency with Tables 3, 4, Binary-labeled clips correspond to Denom A, and the Speech-usable subset corresponds to Denom B. The SyncNet baseline uses the audio present subset, which is a superset of Denom B.

Although not a clinical prediction or diagnostic accuracy study on real teleconsultations, reporting aligns with applicable nonclinical items from TRIPOD + AI and STARD-AI for transparency (Collins et al., 2024; Sounderajah et al., 2025), while CONSORT-AI and SPIRIT-AI are not claimed because they target interventional trials and protocols (Liu et al., 2020; Rivera et al., 2020).

## Results and descriptor selection on the DFRW dataset

We report DFRW dataset performance using ROC and DET curves, AUC, partial AUC in the low false positive region, and fixed low false alarm operating points, complemented by the operational metrics p_df, p_real, Δp, and PR. This reporting supports comparability across articles and makes the low false alarm tradeoff explicit (Fawcett, 2006; Martin et al., 1997). Evaluation denominators and modality-specific subsets are summarized in Table 2 and encoded consistently as Denom A for the full binary-labeled test split and Denom B for the speech-usable subset used by audiovisual descriptors.

For each descriptor d, we compute a clip score s_d and sweep the threshold τ to obtain ROC curves (TPR *versus* FPR) and DET curves, with emphasis on the low error region (Fawcett, 2006; Martin et al., 1997). We report AUC and pAUC restricted to FPR≤1% and FPR≤0.1% as primary criteria for low false alarm ranking (McClish, 1989; Ma et al., 2013), together with TPR at FPR = 1% and FPR = 0.1%, which correspond approximately to 10 and 1 false alarms per 1,000 authentic clips. To quantify operational workload, we report Esc_per_1000 = 1,000 (π_real FPR + π_df TPR) and evaluate base rate sensitivity for π_d ∈ {0.1%, 1%, 5%}. We parameterize escalation cost as Cost_per_1000 = Esc_per_1000·t_esc·c_min to keep estimates transferable across deployments. Thresholds are selected on the validation split, as described in the Materials and Methods section. All curves and metrics are computed on the held-out test split, with bootstrap confidence intervals reported in the Results section (Efron and Tibshirani, 1994).

To remove ambiguity, we report three regimes. First, a reference unconstrained regime selects τ by maximizing Δp over target p_real in the range 1%–30%. This regime is reported only to characterize separability, including cases where p_real approaches 30%. Second, a single descriptor-constrained regime enforces p_real ≤ 20% on validation, and atlas inclusion is evaluated at this operating point by additionally requiring p_df ≥ 30% and either Δp Δ 0.15 or PR ≥ 1.5. It is reported in Table 4 and in the low-FPR portions of the ROC and DET curves. As above, the p_df ≥ 30% requirement applies to motion and flow descriptors at the p_real ≤ 20% operating point; audiovisual descriptors at a fixed extremely low FPR (e.g., ≥ t_AV at p_real = 5.1%) are admitted via high PR and low-FPR sensitivity. See the Table 4 note. Third, a security screening regime fixes FPR at 1% and 0.1% and reports the corresponding TPR, with pAUC at FPR ≤ 1% and FPR ≤ 0.1% treated as primary operational criteria. Fusion results are reported separately for the constrained and security regimes because combining complementary cues can satisfy low-FPR constraints more effectively than any single descriptor.

**FIGURE 1:**
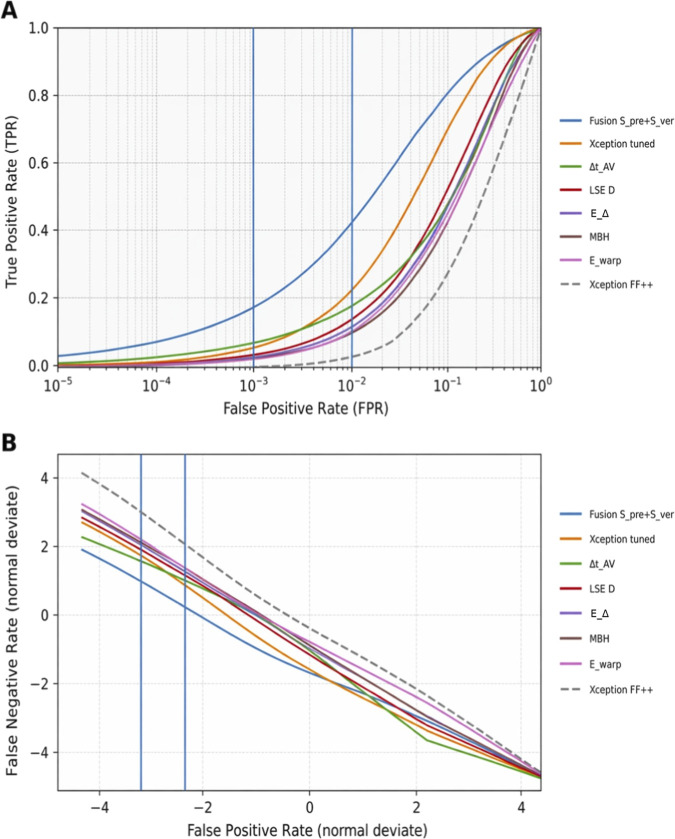
**(A)** ROC curves for the top descriptors, baselines, and the composite fusion on the DFRW test split. **(B)** DET curves emphasizing the extremely low false alarm region on the DFRW test split.

Table 3 reports the unconstrained regime. Table 4 reports the constrained regime with α restricted to 1%–20% on validation (constrained regime only). The corresponding threshold τ is fixed from validation and applied to the test split. Therefore, the observed test p_real can deviate slightly from approximately 20% due to finite sample effects. All operating points are recomputed on the test split for final reporting. Figure 1A presents ROC curves for the top descriptors and the composite fusion, and Figure 1B presents DET curves highlighting the low false alarm region. Table 5 summarizes AUC, pAUC at FPR≤1% and FPR≤0.1%, and TPR at FPR = 1% and 0.1%, and includes a DFRW-tuned Xception baseline to enable distribution-realistic comparison.

Ranking by low-FPR pAUC identifies E_Δ, MBH, and E_warp as the strongest and most stable motion and flow cues, while LSE D, Δt_AV, and r_F0M provide complementary audiovisual evidence. Δt_AV tends to exhibit particularly low false alarm propensity when audio quality is adequate, reflected in higher pAUC at FPR ≤ 0.1%. Because DET curves show that full range AUC rankings can diverge from low-FPR rankings, we treat pAUC and TPR at fixed low FPR as primary. In the unconstrained regime, E_Δ, MBH, and E_warp reach PR 1.93, 1.83, and 1.80 with Δp in the 0.24 to 0.26 range (Table 3). In the constrained atlas regime, the corresponding operating points yield PR 2.10, 2.03, and 1.95 with Δp in the 0.19 to 0.22 range (Table 4), while LSE D reaches PR 2.20, Δt_AV reaches PR 4.20 at p_real 5.1%, and r_F0M reaches PR 1.80. To make operational validity explicit, we report both constrained results (p_real ≤ 20%) and fixed-FPR results at 1% and 0.1% for each descriptor.

The practical interpretation of the very low false positive operating points is intentionally conservative. At FPR = 0.1%, the benchmark operating point corresponds approximately to one false escalation per 1,000 authentic clips, whereas FPR = 1% corresponds approximately to 10 false escalations per 1,000 authentic clips. The fusion model reaches TPR 18.5% at FPR = 0.1% and TPR 42.0% at FPR = 1%, which means that the system should not be interpreted as a standalone authentication mechanism or as a detector expected to identify most manipulated events at the strictest threshold. Instead, these operating points define a low-burden screening layer intended to identify a subset of higher-risk events for step-up verification while preserving workflow continuity for most authentic teleconsultation segments. In practical terms, the FPR = 0.1% threshold is best suited to continuous background screening, where false alerts must remain rare, whereas the FPR = 1% threshold may be more appropriate for short high-impact checkpoints, such as consent capture, prescribing, identity re-verification, referral authorization, or billing review, where higher sensitivity can justify a higher but still bounded escalation burden. Therefore, the reported performance should be read as evidence of risk-based escalation utility rather than complete manipulation detection.

Because single cues rarely retain high sensitivity at FPR = 0.1% under severe degradations, we evaluate a two-stage fusion. The prescreen score S_pre combines lightweight cues (E_Δ, MBH, J, LSE D) by mapping each score to a calibrated probability via isotonic regression on validation (Guo et al., 2017), converting to log odds z_d, then applying a nonnegative weight constrained logistic model S_pre = σ(b_pre+Σ_d w_d·z_d) with parameters fit on validation and frozen for test. The verification score S_ver augments S_pre with higher cost cues (E_warp and flow inconsistency signals, including RAFTs when reliable) and conditionally adds Δt_AV and r_F0M only when audio is present and meets speech usability criteria U_speech. Missing or unusable audio is treated as unavailable rather than imputed, and the integrity report records modality absence for escalation policy. Fusion is evaluated with ROC and DET curves, AUC, low-FPR pAUC, and TPR at FPR = 1% and 0.1%, with operational interpretation via the cost model. Gains are concentrated in the extremely low-FPR region because complementary cues reduce correlated failures, and conditional gating leverages audiovisual evidence without penalizing silent clips.

**FIGURE 2:**
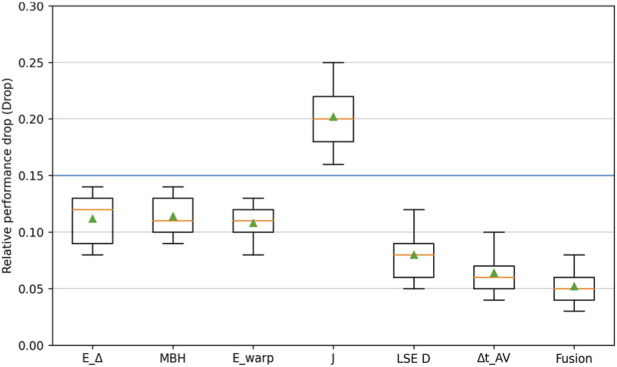
Stability plots showing the distribution of Drop_TPR at FPR = 0.1% across degradation strata.

We replace qualitative stability statements with explicit drop metrics. For partial area under the ROC curve, we define Drop_pAUC(d, k) = 1−pAUC_k(d)/pAUC_ref(d). For fixed operating points, we define Drop_TPR (d, k, α) = 1−TPR_k (d, FPR = α)/TPR_ref (d, FPR = α), where α∈{1%, 0.1%}. For exceedance metrics, we define Drop_p_df (d, k) = 1−p_df_k (d, τ)/p_df_ref (d, τ), computed under the constrained selection rule with p_real controlled by quantile thresholding. A descriptor is considered stable under condition k if the point estimate satisfies Drop_pAUC ≤ 0.15 in the low-FPR region and Drop_TPR at 0.1% ≤ 0.15. We report 95% bootstrap confidence intervals to quantify uncertainty and to highlight borderline strata (Efron and Tibshirani, 1994). Table 6 reports Drop_pAUC across codec, CRF, resolution, recapture, and filter strata, while Figure 2 summarizes the Drop_TPR distributions. 

**TABLE 6:** Quantitative stability under platform degradations based on Drop_pAUC. Relative drops are reported with 95% confidence intervals when available. Each drop cell reports Drop_pAUC for the worst stratum level of the given degradation axis. Stability is satisfied when the point estimate fulfills Drop_pAUC≤0.15. Drop_TPR at FPR=0.1% is summarized separately in Figure 2.

Descriptor	Reference metric	Worst codec drop	Worst CRF drop	Worst resolution drop	Recapture drop	Filter drop	Stable under all axes
E_Δ	pAUC≤0.1%	0.08 (0.05–0.11)	0.12 (0.09–0.15)	0.14 (0.11–0.17)	0.13 (0.10–0.16)	0.09 (0.06–0.12)	Yes
MBH	pAUC≤0.1%	0.09 (0.06–0.12)	0.11 (0.08–0.14)	0.13 (0.10–0.16)	0.14 (0.11–0.17)	0.10 (0.07–0.13)	Yes
E_warp	pAUC≤0.1%	0.10 (0.07–0.13)	0.13 (0.10–0.16)	0.12 (0.09–0.15)	0.11 (0.08–0.14)	0.08 (0.05–0.11)	Yes
J	pAUC≤0.1%	0.18 (0.15–0.21)	0.22 (0.19–0.25)	0.25 (0.22–0.28)	0.20 (0.17–0.23)	0.16 (0.13–0.19)	No
LSE D	pAUC≤0.1%	0.06 (0.04–0.08)	0.09 (0.07–0.11)	0.08 (0.06–0.10)	0.12 (0.09–0.15)	0.05 (0.03–0.07)	Yes
Δt_AV	pAUC≤0.1%	0.05 (0.03–0.07)	0.07 (0.05–0.09)	0.06 (0.04–0.08)	0.10 (0.08–0.12)	0.04 (0.02–0.06)	Yes
Fusion	pAUC≤0.1%	0.04 (0.02–0.06)	0.06 (0.04–0.08)	0.05 (0.03–0.07)	0.08 (0.06–0.10)	0.03 (0.01–0.05)	Yes

We report statistical uncertainty for AUC, pAUC, and fixed point TPR using nonparametric bootstrap on the held-out test split with B = 2,000 resamples, stratified by manipulation family and degradation level, and grouped by identity cluster to account for identity correlations. We compute 95% confidence intervals using both percentile and bias-corrected accelerated methods (Efron and Tibshirani, 1994). Descriptor comparisons in the extremely low false alarm regime use paired bootstrap tests on pAUC at FPR ≤ 0.1% and on TPR at FPR = 0.1%, estimating two-sided p-values from bootstrap replicate differences and controlling multiple comparisons with the Holm adjustment (Holm, 1979). Table 7 reports which performance differences remain statistically reliable under these constraints.

**TABLE 7:** Paired bootstrap comparison tests in the extremely low false alarm regime, with Holm-adjusted p-values.

Metric	Method A	Method B	Δ (A− B)	95% CI for Δ	p-value	Holm-adjusted p-value
pAUC ≤ 0.1%	Fusion S_pre + S_ver	E_Δ	+0.020	(0.015, 0.025)	<0.001	<0.001
pAUC ≤ 0.1%	Fusion S_pre + S_ver	MBH	+0.021	(0.016, 0.026)	<0.001	<0.001
pAUC ≤ 0.1%	Fusion S_pre + S_ver	LSE D	+0.019	(0.014, 0.024)	<0.001	<0.001
TPR @ 0.1%	Fusion S_pre + S_ver	E_Δ	+15.3%	(12.5%, 18.1%)	<0.001	<0.001
TPR @ 0.1%	Fusion S_pre + S_ver	MBH	+15.7%	(12.9%, 18.5%)	<0.001	<0.001
TPR @ 0.1%	Fusion S_pre + S_ver	LSE D	+14.4%	(11.8%, 17.0%)	<0.001	<0.001
TPR @ 0.1%	MBH	E_warp	+0.3%	(-0.5%, 1.1%)	0.450	0.450 (NS)
pAUC ≤ 0.1%	MBH	E_warp	+0.000	(-0.001, 0.001)	0.820	0.820 (NS)

Paired tests are computed via paired bootstrap on the test split. The Holm procedure controls the family-wise error rate across the reported comparisons.

**FIGURE 3:**
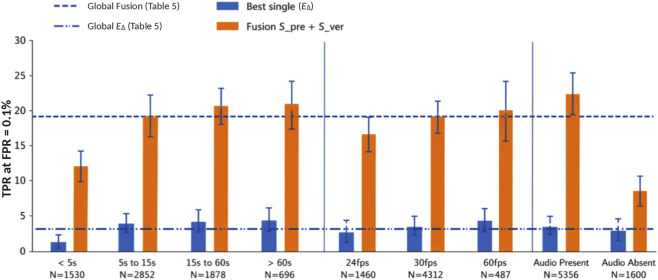
Sensitivity analysis of TPR at FPR = 0.1% across clip length, frame rate, and audio availability for the top single descriptor and the fusion S_pre + S_ver on the DFRW test split. The figure reports stratified low-FPR sensitivity estimates with 95% confidence intervals computed via bootstrap, highlighting how performance changes across deployment-relevant covariates. Audio-dependent cues are computed only on the speech-usable subset defined by U_speech ≥ 0.10.

We assess sensitivity to deployment covariates via stratified analyses over clip length (<5 s, 5 s–15 s, 15 s–60 s, >60 s), frame rate (24fps, 25fps, 30fps, and 60fps), and audio availability (audio present versus absent). Metrics are recomputed per stratum with 95% confidence intervals. Motion cues typically weaken on very short clips due to limited temporal support, flow cues can degrade at low frame rates, and audiovisual cues require usable voiced segments and are evaluated only when audio meets minimum quality criteria. Fusion handles missing audio by gating rather than imputation to avoid penalizing silent clips. Figure 3 summarizes stratum effects by plotting TPR at FPR = 0.1% for the best single descriptor and for fusion. Together with the low-FPR curves, stability drops, and bootstrap intervals, these analyses provide an auditable security evaluation narrative and make explicit whether operational constraints such as p_real ≤ 20% and fixed low-FPR screening points are satisfied.

## Discussion

The DFRW dataset results support the working hypothesis that motion dynamics and audiovisual temporal coherence remain comparatively stable and explainable under recompression, downscaling, filtering, and recapture. The key finding is complementarity between kinematic and cross-modal synchrony cues, motivating an evidence atlas with conservative selection, staged fusion, and explicit abstention because several single cues remain discriminative yet operationally limited by elevated p_real at scale. Operationally, the relevant objective is workload and risk-constrained screening rather than peak separability, and motion and synchrony cues add value because they yield auditable evidence artifacts such as localized motion anomalies, rigid motion warping residual maps, and explicit audio mouth lag curves. In telemedicine and teledentistry, guidance on clinically usable remote audiovisual observation effectively defines the attack surface and integrity requirements (Telehealth.HHS.gov, 2024), while empirical work links diagnostic confidence and concordance to visit quality, implying that integrity failures can affect decision pathways (Demaerschalk et al., 2022; Krupinski et al., 1999). The atlas, therefore, supports verification-oriented integrity flagging without claiming clinical replacement.

From an operational perspective, the reported sensitivity at very low false positive rates should be evaluated against the role of control in the workflow. In routine telemedicine communication, a threshold producing one false escalation per 1,000 authentic clips may be acceptable if escalation only requests a repeated guided capture, secondary confirmation, or delayed review. In contrast, for high-impact actions such as prescribing, consent capture, identity confirmation, referral authorization, certification, or reimbursement, lower sensitivity may still be operationally valuable if the detected cases represent otherwise unrecognized manipulation attempts and the false positive burden remains sufficiently low for human review. Therefore, acceptable sensitivity is not a universal value. It depends on the base rate of manipulation, the harm associated with missed attacks, the time cost of escalation, the availability of second-channel verification, and the clinical or administrative consequence of the action being protected. The present results support use as a risk-based screening and escalation layer, not as a definitive gate that should replace identity verification, provenance controls, clinician judgment, or institutional incident response procedures.

The generalizability of the reported results remains limited to the DFRW dataset and to the stated degradation, modality, and quality strata. Therefore, it should not be interpreted as evidence of equivalent performance across age groups, ethnic backgrounds, skin tones, languages, speech patterns, or medical conditions. This distinction is particularly important in telemedicine because the proposed descriptors rely on facial motion, landmark stability, mouth-opening dynamics, optical flow, and audiovisual synchrony. These signals may be influenced not only by synthetic manipulation but also by natural physiological variability, age-related differences in facial motion, neurological or neuromuscular impairment, facial palsy, craniofacial or dental conditions, voice disorders, respiratory limitations, and post-surgical changes. Consequently, the proposed control should be considered a screening and escalation aid that requires subgroup-specific validation before use in heterogeneous clinical populations.

To address this limitation, future validation should use prospectively collected or ethically curated teleconsultation data with documented demographic and clinical covariates, while maintaining compliance with privacy, consent, and data minimization requirements. At minimum, validation should report subgroup-specific AUC, partial AUC for FPR≤1% and FPR≤0.1%, TPR at FPR = 1% and FPR = 0.1%, calibration error, abstention rate, false alarm rate, and confidence intervals stratified by age band, sex, ethnicity or skin tone where such variables are legally and ethically available, language, speech usability, video quality, lighting conditions, device class, and clinically relevant conditions affecting facial motion or voice. Statistical assessment should include interaction analysis or stratified bootstrap comparisons to determine whether performance degradation in any subgroup exceeds a predefined acceptability margin. Until such evidence is available, threshold policies should remain conservative and should include human review, repeated guided capture, or alternative identity verification in cases with low signal reliability or potentially confounding clinical conditions.

We position integration as a safety-oriented escalation workflow, not a binary gate, addressing impersonation, symptom evidence manipulation, fabricated consent or identity cues, and instruction tampering. We treat the integration patterns below as a deployment hypothesis rather than a validated clinical recommendation. One plausible insertion point is a media gateway or recording service, such as a WebRTC SFU or multipoint control unit (MCU), where an integrity service subscribes to streams and processes buffered segments to emit near-real-time risk signals and evidence artifacts, subject to prospective evaluation and site-specific governance. In an illustrative inline configuration, the service can perform continuous prescreening during the call and emit risk alerts to the session interface or security console. Because Stage 2 verification has a median GPU latency of approximately 2.5 s per 2.0 s window, Stage 2 is designed as buffered asynchronous verification that reports *post hoc* on the analyzed segment rather than as a synchronous gate on the live stream. In an asynchronous configuration, the service can analyze recordings *post hoc* and attach an integrity report to documentation. For high-impact steps such as consent capture, prescribing, or reimbursement, the service can be invoked at explicit checkpoints to trigger additional verification. The two-stage control instantiates Stage 1 prescreening with lightweight, platform-robust cues (E_Δ, MBH, E_fb, J, R_bg_face, and LSE D or LSE C when speech is usable) and Stage 2 verification only for elevated risk segments using costlier analyses (RAFT-based flow errors and EPE, E_warp, longer window Δt_AV and r_F0M, and optional bitstream cues). Prescreening runs on 2.0 s windows with a 1.0 s stride to provide at least 1 Hz updates and should complete within the stride to avoid backlog, while verification can budget 3.0 s–10.0 s to support step-up challenges that pause only high-impact actions.

Microbenchmarks validate feasibility on a reference system (Intel Core i9 13900K, 64 GB RAM, NVIDIA RTX 4090 24 GB, Ubuntu 22.04, PyTorch 2.1, OpenCV 4.8 CUDA). Timing uses 2.0 s windows with a standardized 60-frame workload at 30fps and 720px short side resizing, reporting median and p95 over N = 1,000 randomized DFRW test windows in Table 8, with full logs in the harness. Stage totals are measured from start to finish and include orchestration overhead such as decoding, tensor preparation, device transfer, and report assembly, while individual block rows report isolated microbenchmarks and are therefore not required to sum exactly to totals. Stage 1 totals are 215 ms median GPU and 840 ms median CPU, with CPU p95 915 ms, meeting the 1.0 s stride. Stage 2 totals are 2450 ms median GPU with p95 2650 ms, within the 3.0 s–10.0 s verification budget. In the intended deployment, Stage 2 runs asynchronously on buffered segments and does not block the live call. It emits a delayed alert and evidence for the corresponding segment while the conversation proceeds normally. Only high-impact workflow steps such as consent capture, prescribing, or reimbursement are gated on Stage 2 completion. Stage 2 is not intended for CPU real-time use. Translating p95 to capacity, Stage 1 yields floor (1000 ms/240 ms) = 4 concurrent sessions per GPU under 1 Hz sequential evaluation and approximately one session per CPU worker at p95, while Stage 2 supports approximately 60,000 ms/2,650 ms ≈ 22 verifications per minute per GPU. With a rate limit of one escalation per session per minute, this corresponds to approximately 22 concurrently active sessions per GPU at p95.

**TABLE 8:** Latency and throughput microbenchmark results for the two-stage control on 2.0 s windows (60 frames).

Block	Win	CPU med/p95 m	GPU med/p95 m
E_Δ	A	12/18	2/3
MBH	A	385/420	45/52
LSE D	B	95/110	18/22
Stage 1 overhead	C	340/365	145/160
Stage 1 total	A, C	840/915	215/240
RAFT flow inference	D	N/A	1850/1950
E_warp and consistency	D	450/480	55/65
Δt_AV extended lag	A	120/140	35/45
Stage 2 total	D	N/A	2,450/2,650

A: 2.0 s, 60 frames, Face ROI 224px. B: 2.0 s, 16 kHz plus Face crop 96px. C: Face detection and ROI tracking overhead. D: 60 frames, Face ROI approximately 512px mapped for flow. Stage 1 total and Stage 2 total are measured from start to finish and include non-itemized overheads, so block rows are not strictly additive.

The control explicitly supports abstention when reliability is low, including unstable face detection or tracking beyond the thresholds defined in the Materials and Methods section, small face ROI, excessive occlusion, unresolved multi-face cases, or absent or unusable audio for audio-dependent metrics. Abstention is treated as elevated uncertainty, triggering safe actions such as prompting capture improvements, requesting a repeated guided short capture, switching modality, or escalating to manual verification. When speech is absent, the system switches to motion-only evidence and disables audio-based paths to avoid spurious LSE or Δt_AV signals.

Evidence is mapped to three states: clear with standard workflow, escalate requiring step-up verification such as challenge response, secondary channel confirmation, or a second capture, and block pausing high-impact actions for manual review when repeated verification remains strongly suspicious. Here, Block applies to the targeted action in the workflow user interface (UI), not to conversational turn-taking. The audiovisual stream remains continuous while verification completes. Policies are parameterized by p_real budgets and risk context, with stricter thresholds for prescribing, consent, and billing than for routine counseling, and record an audit trail of thresholds, reliability indicators, and decisions.

The Esc_per_1000 metric should be interpreted as an operational planning measure rather than as a clinical outcome. For example, at FPR = 0.1% and a low manipulation base rate, most escalations will still be false alerts because authentic encounters dominate the workflow volume. This is acceptable only if escalation is lightweight, auditable, and proportionate to the protected action. Conversely, higher sensitivity at FPR = 1% may be preferable for high-risk administrative or clinical checkpoints if the institution can absorb approximately 10 false escalations per 1,000 authentic clips. Threshold selection should therefore be local and policy-driven: lower FPR thresholds are suitable for continuous background screening, whereas higher sensitivity thresholds may be reserved for short, high-impact checkpoints such as consent recording, controlled symptom capture, prescribing, billing, or identity re-verification. Outputs are delivered as a structured integrity report rather than a single score, with machine-readable JSON and a human summary. The report records metadata, timestamps, processing versions, decision state, abstain reasons, descriptor scores with thresholds, reliability indicators, and links to evidence artifacts such as ROI localized motion heatmaps, warping residual maps, flow consistency summaries, and audiovisual lag curves when speech is usable. The summary states which cues triggered escalation, which were unavailable, and recommended actions, explicitly positioning outputs as integrity risk signals rather than medical inferences.

Teledentistry guidance distinguishes synchronous video encounters from asynchronous store-and-forward interactions and expects documentation of modality, captured elements, transmission, retention, and audit trail, aligning with the proposed evidence artifact logging (American Dental Association, 2023). Formative usability is not evaluated here, and the reporting format is a deployment hypothesis. Future work can assess interpretability and actionability with n = 5 to 10 telehealth clinicians and governance staff without claiming clinical outcomes.

Audio cues require available, usable speech and may fail under missing audio, heavy filtering, or music replacement despite 77% audio presence in the DFRW dataset. Motion and flow cues depend on robust face detection, landmarks, and segmentation and can degrade under occlusions, profile views, extreme motion, multiple faces, blur, and heavy augmented reality overlays, increasing both miss and false alarm risk. The DFRW dataset models platform degradations but remains a snapshot as generators improve temporal stability and synchrony and as antiforensic postprocessing targets temporal cues, motivating periodic atlas reevaluation and dataset updates. OSINT provenance uncertainty further supports conservative deployment with calibrated thresholds, explicit abstention, and structured reporting that routes borderline cases to human review, consistent with broader synthetic content risk reduction guidance emphasizing governance and realistic testing. The contribution is therefore a controllable measurement framework providing stable, interpretable signals for escalation and auditing in high-impact workflows rather than a claim of universal detection against future generators.

## Conclusion and future work

We empirically assessed motion dynamics and audiovisual temporal coherence as explainable integrity signals under distribution-realistic platform processing, combining the DFRW dataset with an operational selection protocol prioritizing low false alarms and degradation stability. This study does not report clinical validation on real teleconsultations. All results are derived from the DFRW benchmark and the stated evaluation protocol. The DFRW dataset comprises 46,371 clips (229.28 h) spanning OSINT content and controlled generated plus systematically degraded variants emulating dissemination chains. Accordingly, the reported low-FPR results should be interpreted as evidence for a conservative escalation mechanism with limited but operationally meaningful sensitivity, rather than as evidence of complete manipulation detection in real telemedicine practice.

Results indicate that motion and temporal cues remain informative under recompression, downscaling, filtering, and recapture, where many spatial cues degrade. Key motion descriptors include E_Δ, MBH, and E_warp, consistent with persistent kinematic coherence and nonrigid warping weaknesses in many manipulation pipelines. Key audiovisual descriptors include LSE D, Δt_AV, and r_F0M, with Δt_AV exhibiting particularly low p_real and high PR when audio is usable. Because single cues can yield operationally excessive p_real despite favorable separation, deployment is framed around multi-cue composites and staged decision logic. Under the criteria p_df ≥ 30%, p_real ≤ 20%, Δp ≥ 0.15 or PR ≥ 1.5, and stability defined as a relative drop ≤0.15, that is ≤15%, the p_df ≥ 30% threshold applying to motion and flow descriptors at p_real ≤ 20%, while audiovisual descriptors at a fixed extremely low FPR are admitted via high PR and low-FPR sensitivity) we recommend a core composite {E_Δ, MBH, J, E_warp, LSE D, Δt_AV} supporting a two-stage architecture with lightweight prescreening followed by targeted verification using higher fidelity flow and temporal alignment, with calibrated uncertainty and abstention for analyst-facing workflows.

Future work should include versioned updates of the DFRW dataset covering emerging diffusion-based and hybrid generation families, as well as adversarial postprocessing strategies specifically targeting temporal cues. Further methodological development should also address reliability calibration and uncertainty quantification, with explicit abstention mechanisms for cases affected by uncertain OSINT provenance, heterogeneous capture conditions, or insufficient signal quality. Multimodal extensions should be evaluated together with robustness under audio-absent conditions, including adversarial speech suppression or music-masking scenarios that force the system to rely primarily on motion-based evidence. Prospective evaluation on real teleconsultations should be conducted under appropriate ethical, legal, and governance safeguards. Such evaluation should explicitly include subgroup validation across age groups, ethnic backgrounds or skin tone categories where these variables are legally and ethically available, languages, speech characteristics, device and lighting conditions, and medical conditions affecting facial motion, articulation, voice production, or head movement. The purpose of this future validation should be to quantify workflow impact, subgroup robustness, calibration, abstention behavior, and false alarm burden without making diagnostic claims. The DFRW dataset operates under an internal immutable manifest with clip identifiers and SHA256 hashes to ensure deterministic evaluation and provenance tracking. However, owing to the inclusion of OSINT assets subject to third-party terms and privacy constraints, neither the raw media nor the derived feature sets are redistributed. The reported metrics and degradation protocols are documented to support methodological transparency without dataset release.

## Data Availability

The datasets presented in this article are not readily available because the DFRW dataset contains OSINT assets subject to third-party licensing and privacy restrictions and, therefore, cannot be publicly released. The internal immutable manifest (clip identifiers and SHA256 hashes), degradation protocols, and full evaluation methodology are described to ensure reproducibility. Aggregated descriptor-level statistics and evaluation scripts are available from the authors upon reasonable request. Requests to access the datasets should be directed to Karol Jędrasiak, kjedrasiak@wsb.edu.pl.
